# Systematic analysis of the factors that adversely affect the rate of cell accumulation in mouse embryos during their culture *in vitro*

**DOI:** 10.1186/1477-7827-12-35

**Published:** 2014-05-08

**Authors:** Xing L Jin, Chris O’Neill

**Affiliations:** 1 Developmental and Regenerative Medicine, Kolling Institute for Medical Research, Sydney Medical School, University of Sydney, Sydney, NSW 2065, Australia

**Keywords:** Reproductive techniques, Zygote, Blastocyst, Embryotrophins, Nutrition, Cell hypoxia

## Abstract

**Background:**

Retarded embryo growth is a pervasive effect of culture in vitro.

**Methods:**

A systematic analysis of the interactions between media design, embryo culture density, oxygen tension, amino acids, trophic ligands and the genetic background of the mouse on embryo growth rates in vitro was performed.

**Results:**

Growth retardation of mouse zygotes was greater in 20% O_2_ than 5%, a sequential media design was superior to static simple media designs, but the supplementation of simple media with mixed amino acids mitigated this difference. There was a beneficial effect of communal culture in small volumes, and supplementation with a trophic ligand (Paf) further enhanced growth rates. For hybrid strain zygotes (B6CBF1) communal culture in KSOM media supplemented with amino acids, albumin and Paf under 5% O_2_ resulted in complete rescue of their rate of accumulation of cells and blastocyst formation. Inbred strain (C57BL6/J) zygotes, however, still showed some retardation of development under these conditions. The additional supplementation of media with another trophic ligand (IGF1) showed a further additive beneficial effect on development of inbred strain embryos but they still showed a growth deficit of ~ 23% cell number. The results show that optimising the interactions between a range of culture conditions and media design can rescue hybrid strain embryos from a retarded rate of cell proliferation caused by culture in vitro, but this was incomplete for the B6 strain.

**Conclusions:**

The results indicate that the growth requirement of embryos in vitro varies depending upon their genetic background and provide models for the further genetic analysis of embryo growth.

## Background

The culture of the preimplantation embryo is central to most forms of reproductive technology and assisted reproduction. From the earliest days of development of this technology the retarded rate of development of the embryo in vitro compared to those growing in vitro has been a pervasive feature of the technology [1,2].

Recent advances in media design have resulted in improvements in the viability of the resulting embryos, yet the developmental potential of embryos created by assisted reproductive technologies is still regarded as a major limiting factor to success. Advances include the development of sequential media systems in an attempt to more closely replicate the dynamic metabolic requirements of the embryo at different stages of development [3]. An alternative approach has been the systematic empirical development of more optimised media, as exemplified by KSOM [4]. The supplementation of media with mixed amino acids [5,6] provides further benefits, and the advantage of low oxygen concentration has long been recognised [7-10]. There is also evidence for a beneficial role of communal culture of embryos [11-13] to maximise the actions of autocrine tropic ligands [14-16]. This can also be partially achieved by supplementation of media with exogenous ligands [11,17,18]. It is likely that there is a significant genetic component to the embryo’s capacity to develop successfully. There is much anecdotal evidence for marked differences in development in vitro of embryos from mouse strains of different genetic backgrounds. The inbred C57BL/6 J (B6) strain is particularly sensitive to culture in simple defined media, compared to the robust growth response of a hybrid strain [19]. Of interest, the release of putative trophic ligands may also be associated to the genetic background in mice [20-22].

The last decade has seen marked improvements in media and culture system design and this has been associated with consistent improvements in outcomes of assisted reproductive technologies. Yet most studies have looked at variables in isolation and there is only limited understanding of the potential beneficial interactions between the wide range of variables affecting embryo growth rates in vitro. The aim of this study was to determine whether beneficial or deleterious interactions exist between these factors on embryo growth in vitro. The results define the range of conditions required for complete rescue of the growth rate of mouse hybrid strain embryos, and points to areas for further development for strains that are most sensitive to the effects of culture.

## Methods

### Animals

The use of animals was in accordance with the Australian Code of Practice for the Care and Use of Animals for Scientific Purposes and was approved by the Institutional Animal Care and Ethics Committee. Inbred strain (C57BL/6 J, B6) and hybrid strain (C57BL/6 J X CBA/He, B6CBF1) mice were used in experiments. Animals were housed and bred in the Kearns Research Laboratory, St Leonards, NSW, Australia. All animals were under 12 h light: 12 h dark cycle and had access to food and water ad libitum. Six week old females had ovulation-induced by intraperitoneal injection of 5 IU equine chorionic gonadotrophin (Folligon, Intervet International, Boxmeer, The Netherlands) followed 48 h later by 5 IU human chorionic gonadotrophin (hCG, Chorulon, Intervet). Females were paired with males of proven fertility. Pregnancy was confirmed by the presence of a copulation plug the following morning (day 1). Embryos were collected at various times after hCG (h post-hCG) as shown in results.

### Mouse embryo collection and culture

Zygotes were collected from the reproductive tract 18 h post-hCG in Hepes-buffered human tubal fluid medium (Hepes-HTF) [14] and freed from their cumulus cells by brief exposure to 300 IU hyaluronidase (Sigma Chemical Company, St Louis, MO). Two-cell embryos were collected at 40 h post-hCG. After thorough washing in Hepes-HTF, embryos were cultured in human tubal fluid medium supplemented with glutamine and EDTA (GE-HTF) [14], KSOM [4], or a proprietary human ART media (Sydney IVF medium suite, Cook Australia, Limited) [23]. All components of GE-HTF and KSOM media were tissue culture grade (Sigma). Amino acid supplementation was by the addition of 1 ml MEM amino acid solution (Sigma, cat#11130051) and 0.5 ml MEM non-essential amino acid solution (Sigma Cat#11140050) to 50 ml of media. GE-HTF and KSOM was supplemented with 3 mg bovine serum albumin (BSA)/mL (crystallised, Sigma). In some experiments media was supplemented with the embryotropins Paf (1-*o*-alkyl-2-acetyl-*sn*-glycero-3-phosphocholine) (Sigma) or IGF1 (recombinant Insulin-like Growth Factor-I from mouse, Life Technologies) [14]. Sydney IVF media suite (sIVF) is a sequential media design with two formulations, sIVF cleavage medium (K-SICM-50) and Blastocyst medium (K-SIBM-50) and each is supplemented with human serum albumin. Zygotes were cultured in K-SICM-50 for 48 h (or after 28 h for culture of 2-cell embryos) followed by transfer to K-SIBM-50 for a further 48 h.

Embryos were cultured individually or in groups of 10 in 10 μL drops in 60-well plates (LUX 5260, Nunc, Naperville, IL) overlaid by approximately 2 mm of heavy paraffin oil (Sigma). Culture was performed at 37°C in 5% CO_2_ in either air (20% O_2_) or 5% O_2_, 90% N_2_ for the periods indicated in individual experiments.

### Developmental outcomes

In some experiments embryos were analyzed at a number of time points and cell counts performed to assess the rate of accumulation of cells in the embryo over time. Up to the uncompacted 8-cell stage cell counts could be readily performed by observation by dissecting microscope, from the time of compaction cell counts were performed on cohorts of embryos, which were fixed in 2% (w/v) paraformaldehyde (Sigma) in phosphate buffer saline (PBS) (Sigma) for 30 min and stained with 10 μg/mL Hoechst 33342 (Sigma) in PBS. The stained nuclei were visualised and counted. The proportion developing to morphological blastocysts and hatching blastocysts was recorded. The number of nuclei that showed normal morphology or punctuate or fragmented structure (indicative of degeneration) was recorded.

### Statistical analysis

The statistical analysis was performed with SPSS for Windows (Version 19.0, SPSS Inc., Chicago, IL, USA). The cell number was quantitatively analysed by Univariate analysis of variance. In the model, cell number was the dependent variable, test treatments the independent variables and experimental replicate was incorporated in the model as a covariate. Test of main factor effects and interaction effects was performed. Difference between individual treatments was by multiple comparisons using the least significance difference test. The development rates of blastocysts and hatching blastocysts was assessed by binary logistic regression analysis.

## Results

**Figure 1:**
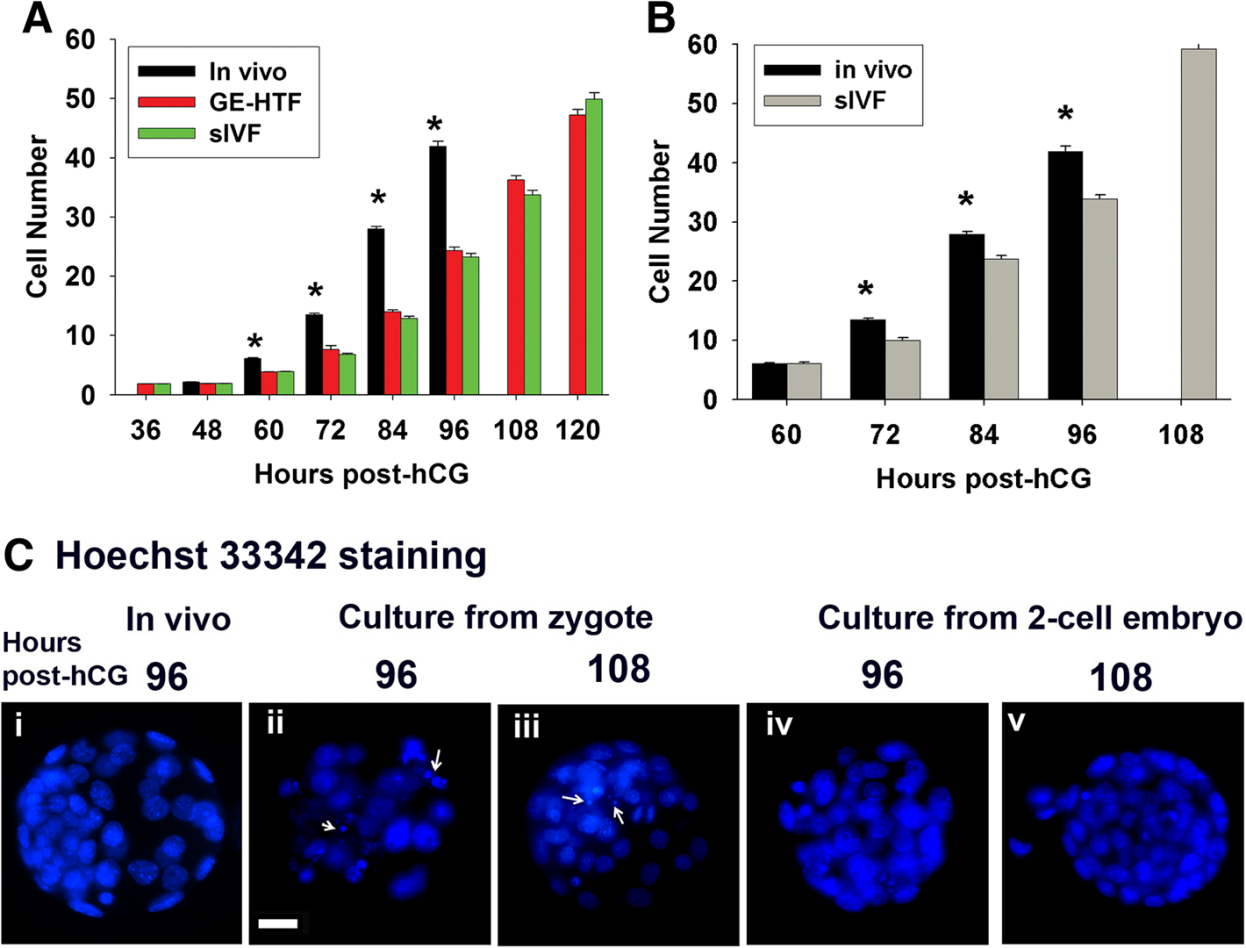
**The effects of embryo culture on developmental viability. (A)** Hybrid strain (B6CBF1) zygotes collected 20 h post-hCG or **(B)** 2-cell embryos 44 h post-hCG were cultured as 10 embryos in a drop of 10 μL GE-HTF medium or sIVF media under 5% CO_2_ in air (20% O_2_). The cell number of the cultured embryos and the embryos freshly collected from reproductive tracts at a 12 h interval from **(A)** 36 h post-hCG or **(B)** 60 h was recorded. Each time point is the record of least 80 embryos from 3 independent replicates. *p < 0.001, compared to the corresponding stage of cultured embryo. **(C)** Representative images of embryos stained with Hoechst 33342 and examples of fragmented nuclei shown with arrows.

The rate of accumulation of cells into hybrid strain preimplantation embryos was faster during development in the reproductive tract compared to during culture in vitro from the zygote stage (Figure 1A & 1C). This difference in growth rates was obvious as early as 60 h post-hCG (42 h after culture commenced). The rate of accumulation of cells in vitro was not different for two media formulations (GE-HTF and sIVF). It was not a result of a block to cell division at any particular stage of development but rather resulted from a persistent pattern of slower cell-doubling time from 60 h post-hCG (after the 2-cell stage was reached). When embryos were collected at the mid 2-cell stage the rate of accumulation of cells within embryos was still slower during culture in sIVF media than in the reproductive tract (Figure 1B & 1C), but this reduction was not as great as occurred when culture commenced at the zygote stage (p < 0.001). The deficit in cell number in embryos commencing culture at the 2-cell stage was not evident until 72 h post-hCG.

**Figure 2:**
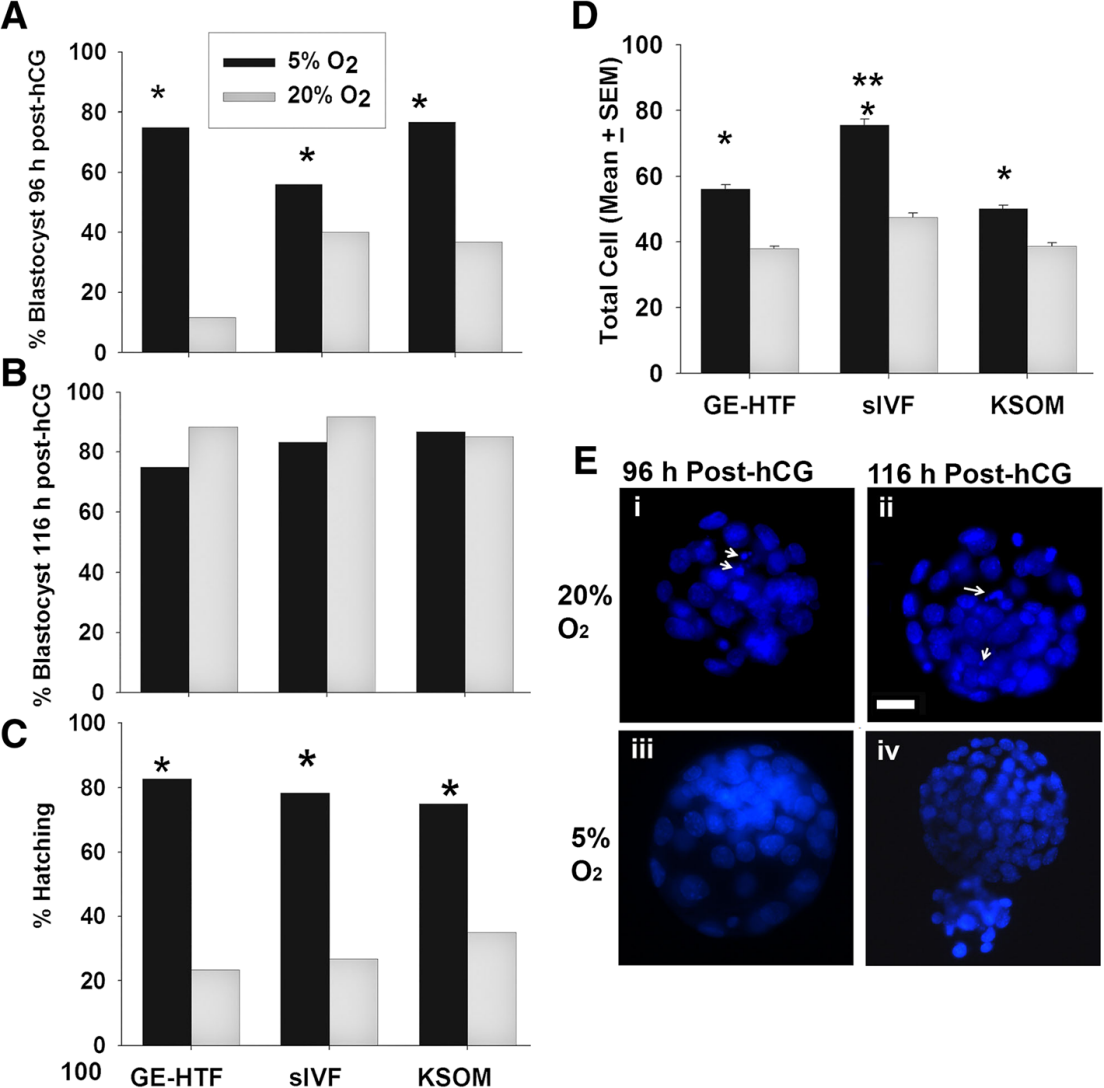
**The effect of O**_**2 **_**tension on growth rate.** Hybrid strain zygotes were cultured as 10 embryos in a drop of 10 μL GE-HTF medium, sIVF or KSOM media. The rates of blastocyst formation **(A)** 96 h and **(B)** 116 h post-HCG, **(C)** the rate of hatching blastocysts and **(D)** the cell number in the resultant blastocyst (116 h post-hCG) were analysed. Each treatment contained at least 80 embryos from two independent replicates. *p < 0.001, compared to the embryos cultured in 20% O_2_. **p < 0.001, compared to modHTM or KSOM media in 5% O_2_. **(E)** Representative images of embryos developed in different O2 tension stained with Hoechst 33342 and examples of fragmented nuclei shown with arrows.

Oxygen tension is commonly considered an important cause of the reduced rate of development of embryos in vitro. The results in Figure 1 were achieved in 20% O_2_. Growth rates were next compared at 5% or 20% O_2_ using hybrid strain (B6CBF1) zygotes cultured in groups of 10 embryos in 3 different media formulations (GE-HTF, sIVF, KSOM). More embryos developed to morphological blastocysts at 96 h post-hCG in 5% O_2_ than 20% O_2_ in each of the three media formulations tested (p < 0.001), however, there were fewer blastocysts in sIVF media at 5% O_2_ than the other two media (p < 0.05) (Figure 2A). By 116 h post-hCG there was no effect of O_2_ tension on the proportion of zygotes that were morphological blastocysts (Figure 2B), but significantly more blastocysts had commenced hatching in low O_2_ tension in all media formulations (P < 0.001) (Figure 2C). Blastocysts (116 post-hCG) cultured in 5% O_2_ had more cells than 20% O_2_, and this increase was greater in sIVF media than either GE-HTF or KSOM (Figure 2D and 2E) (p < 0.001).

**Figure 3:**
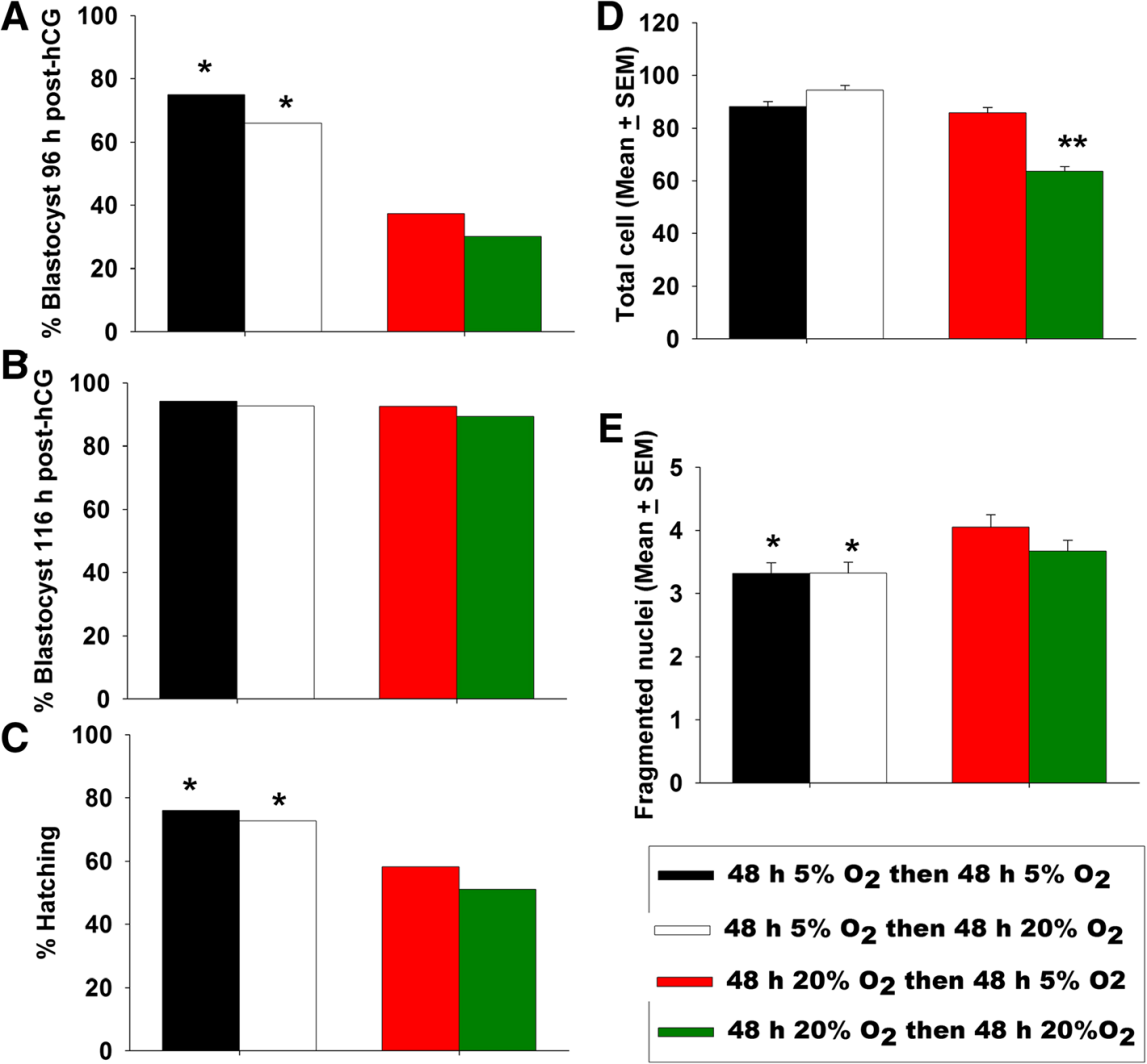
**The effect of duration and periods of changes in O**_**2 **_**tension growth rates in vitro.** Hybrid strain zygotes were cultured in groups of 10 in 10 μL of sIVF cleavage medium in 5% or 20% O_2_ for 48 h. Each treatment was divided into two subgroups and cultured in sIVF blastocyst medium for a further 48 h in 5% or 20% O_2_. The rate of blastocyst formation **(A)** 96 h post-hCG and **(B)** 116 h post-hCG, and the **(C)** the rate of blastocyst hatching, and **(D)** the total numbers of cells, **(E)** fragmented nuclei in the resultant 116 h post-hCG blastocyst was analysed. The results are representative of at least 100 embryos for each treatment from three independent replicates. *P < 0.001, compared to the corresponding treatment group of embryo that was cultured in different O_2_ tension. **p < 0.001, compared to the embryos that were cultured in 5% O_2_ for 48 or 96 h.

The metabolic requirements of the embryo vary considerably during early embryo development so it was of interest to assess whether the embryo’s response to O_2_ concentration changed during different periods of development. We therefore divided the period of culture of zygotes into two 48 h periods and the embryos were exposed to either 5% or 20% O_2_ at each of these periods in sIVF media (Figure 3). The combinatorial arrangements of O_2_ exposure had a significant impact on the rate of development to blastocysts 96 h after hCG. Culture in 20% O_2_ for the first 48 h reduced development (p < 0.001) and there was a compound adverse effect if culture then continued in 20% O_2_ compared to transfer to 5% O_2_ (Figure 3A). By 116 h post-hCG this effect was not obvious for the proportion of embryos reaching blastocysts (Figure 3B) but there was an effect on those hatching (p < 0.001) (Figure 3C). Culture in 5% O_2_ for the first 48 h improved outcomes over culture in 20%, but transfer to different O_2_ concentration in the second 48 h did not further influence this outcome. Of those achieving the blastocyst stage, culture in 5% O_2_ for the first 48 h had an overall beneficial effect on the number of the cells, but for those cultures in 20% for the first 48 h, subsequent transfer to 5% O_2_ could entirely compensate for this (Figure 3D). Low O_2_ in the first 48 h caused a small significant reduction in the number of nuclei showing signs of degeneration (Figure 3E) but there were no significant statistical interaction effect (p > 0.05) of O_2_ tension in the second 48 h of culture. The results demonstrate complex interactions between the temporal profile of O_2_ exposure and developmental outcomes, but clearly show that maintenance at 5% O_2_ for the duration of culture has the best outcomes by all measures.

**Figure 4:**
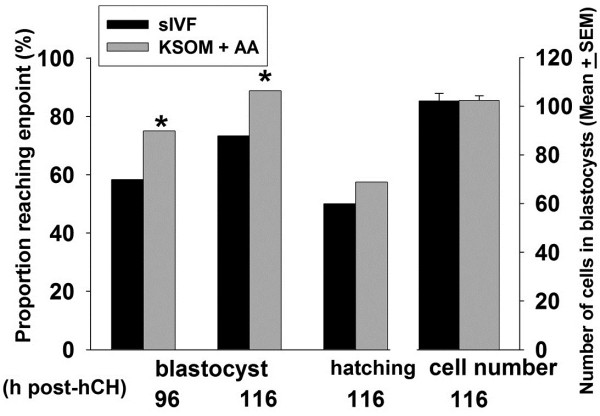
**The effects of supplementation of media with mixed amino acids on growth rate of zygotes.** Hybrid strain zygotes cultured in groups of 10 in 10 μL GE-HTF or sIVF media under the 5% oxygen tension. The developmental outcomes were assessed at 96 h for blastocyst formation and 116 h for the rates of blastocyst formation and hatching, and the number of the cells accumulated in the resultant blastocyst. At least 100 embryos for each medium from 3 independent replicates were tested. *P < 0.001, compared to embryos cultured in sIVF media.

We were surprised by the deficit in cell numbers in KSOM media compared to sIVF media following culture in 5% O_2_ (Figure 2). The major differences between these media are that sIVF media is a sequential media and contains mixed amino acids. Addition of mixed amino acids to KSOM is reported to improved culture outcomes [24] and when added to KSOM in this study amino acids increased the rate of development to blastocysts at 96 and 116 h and the rate of hatching, compared to sIVF media (p < 0.05). The resulting blastocysts had a similar total cell number after culture in either formulation (Figure 4).

**Figure 5:**
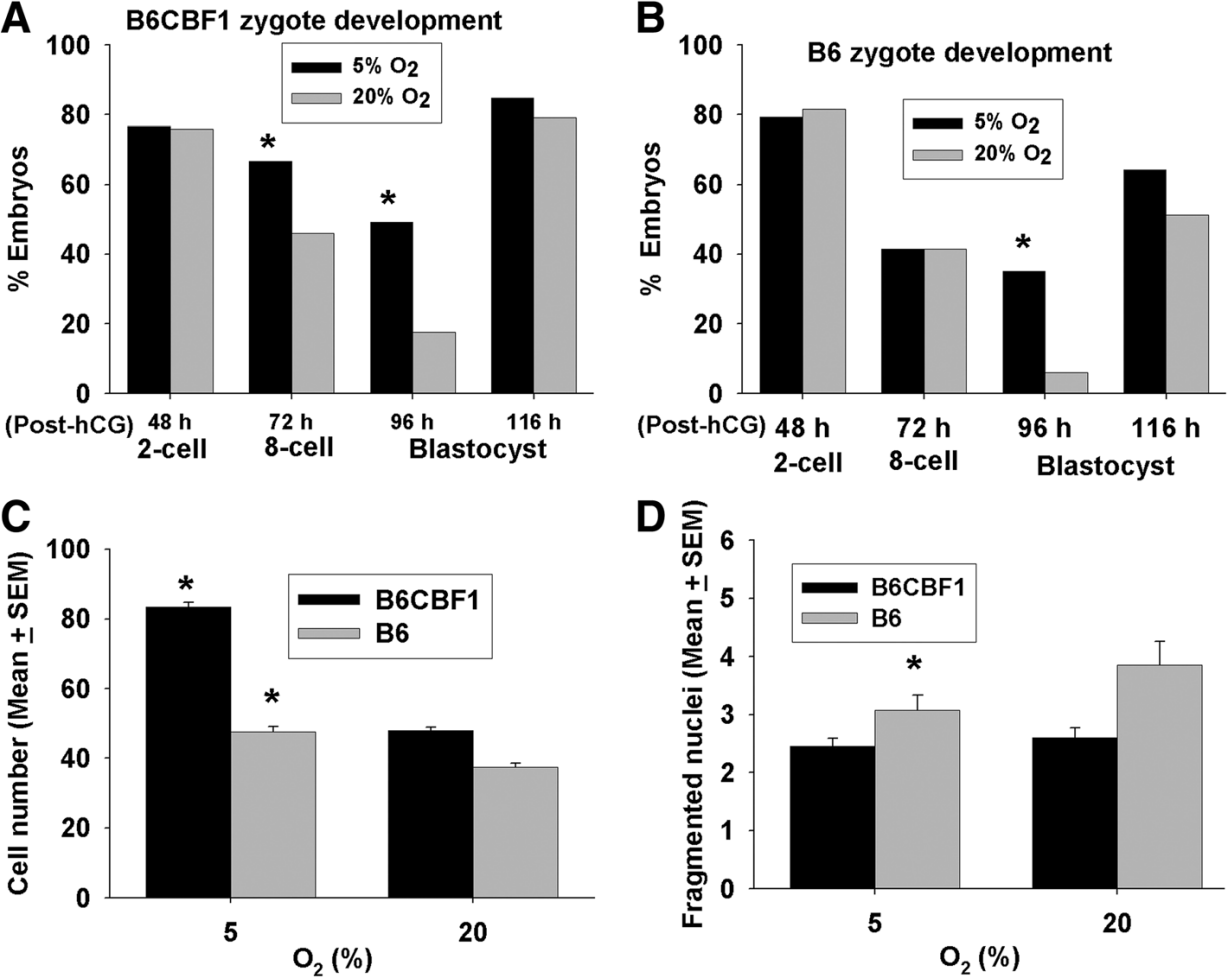
**The effects of O**_**2 **_**tension and genetic background on growth rates in vitro.** The effect of 5% or 20% O_2_ on the proportion of **(A)** hybrid strain and **(B)** B6 mouse zygotes cultured as groups of 10 in 10 μL sIVF media achieving developmental landmarks at 48, 72, 96 and 116 h post-hCG. **(C)** the total number of cells in the resultant blastocysts, and **(D)** the number of these cells with fragmented nuclei in the resultant blastocyst 116 h post-hCG were assessed. At least 200 hybrid strain and 130 B6 embryos from 4 independent replicates were tested. *p < 0.01, compared to the embryos achieving the same landmarks in 20% O_2_ condition.

The foregoing experiments all involved analysis of B6CBF1 hybrid strain embryos. It also had been shown that some inbred strains (such as B6) show greater sensitivity to the adverse effects of culture than do hybrid strains and some outbred strains [19]. We therefore assessed the extent to which culture in low O_2_ in sIVF media could compensate for this strain effect. Like hybrid strains (Figure 5A), B6 strain zygotes in 5% O_2_ had better development to blastocysts at 96 h post-hCG than in 20% O_2_ (p < 0.001) but there was no difference evident at 116 h post-hCG (Figure 5B). The trend response for B6 embryos was very similar to hybrid strain embryos, yet the overall rate of development to blastocysts (at both 96 and 116 h) was poorer for B6 zygotes (p < 0.001). Both strains accumulated more cells in 5% O_2_ than 20% (p < 0.001) (Figure 5C) but B6 embryos had more nuclei with degenerate morphology (Figure 5D) at 116 h post-hCG than B6CBF1 embryos at 5%. Thus, low O_2_ increased the developmental capacity of B6 zygotes, but their overall rate of development and their rate of accumulation of cells were still retarded compared to hybrid strain embryos (Figure 5A).

**Figure 6:**
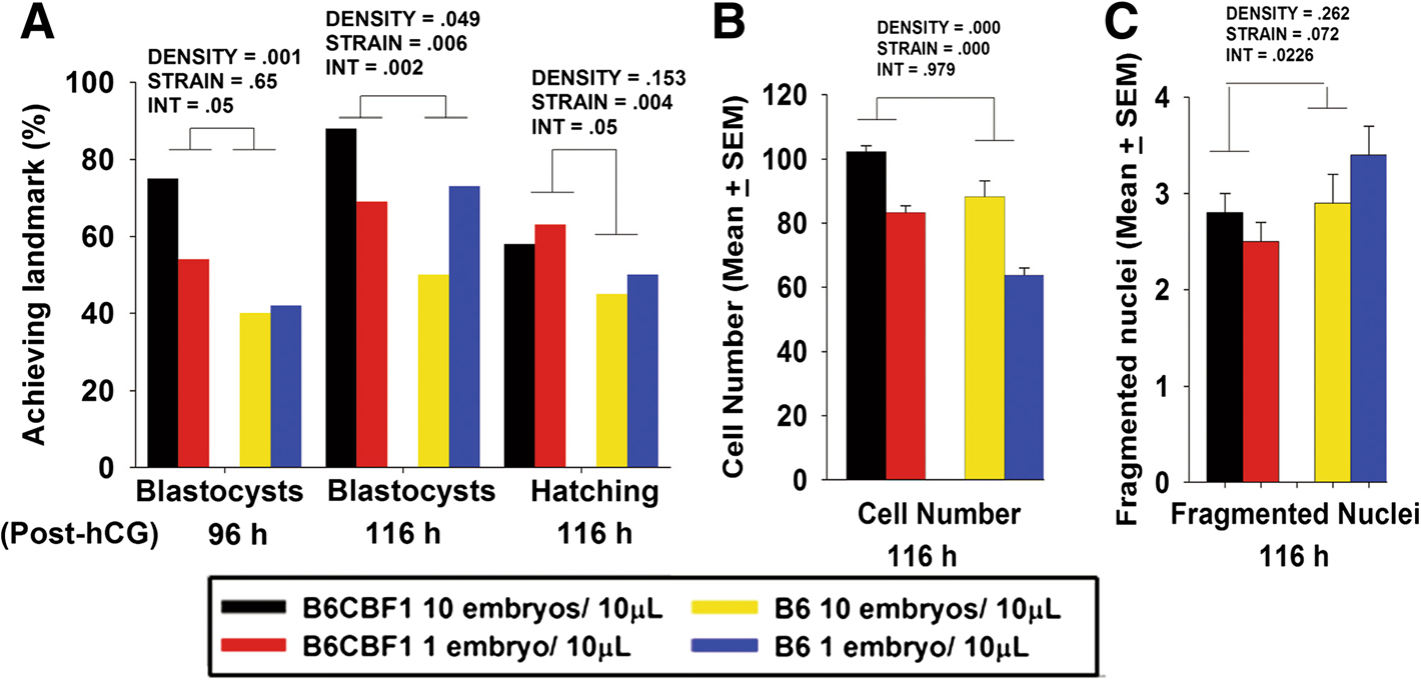
**The effects of embryo density in media on growth rates.** Hybrid strain and B6 mouse zygotes were cultured individually or in groups of 10 embryos in 10 μL sIVF media under 5% O_2_ condition. **(A)** the rates of blastocyst formation at 96 and 116 h post-hCG, and hatching blastocysts, **(B)** the number of the cells in each blastocyst and **(C)** the number of cells with fragmented nuclei were analysed. Each culture group had at least 60 hybrid strain embryos and 50 B6 embryos from 3 independent replicates. Statistically significant effects are shown on the graphs. DENSITY and STRAIN were the dependent variables, and INT (abbreviation of interaction) was the interaction effect between DENSITY and STRAIN.

Successful growth of preimplantation stage embryos depends upon the actions of released autocrine tropic ligands [14,16]. In simple media, embryos have better development during communal culture than when grown individually. To assess whether improved culture conditions removed the need for communal culture, B6 or B6CBF1 zygotes were cultured individually or in groups of ten in sIVF media at 5% O_2_. This analysis showed B6 strain embryos had slower development to each of the three landmarks than did B6CBF1 zygotes (Figure 6A). B6 embryos accumulated fewer cells (Figure 6B), but there was no significant effect of strain on the number of nuclei that showed signs of degeneration (Figure 6C). For B6CBF1 zygotes there was a marked deleterious effect of individual culture compared to communal culture in the rate embryos reached the blastocyst, but not hatched blastocyst stages. Interestingly, this was not the case for B6, with both communal and individual culture doing equally poorly (Figure 6A). There was, however, a similar significant adverse effect of individual culture on the rate of accumulation of cells into blastocysts for both strains (Figure 6B).

**Figure 7:**
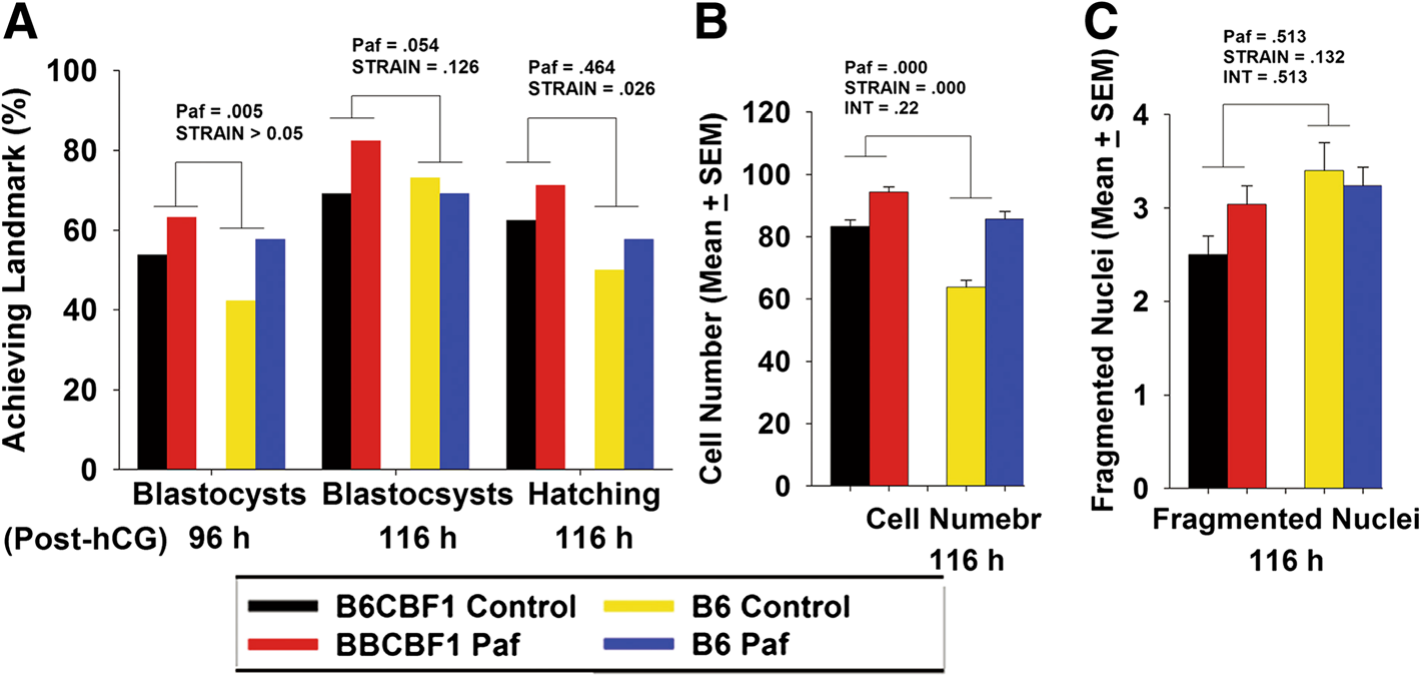
**The effects of exogenous Paf on growth rates.** Hybrid strain and B6 mouse zygotes were cultured individually in a drop of 10 μL sIVF media with (Paf) or without (Control) supplementation with 37.2 nM Paf under 5% O_2_. **(A)** the rates of the blastocyst formation at 96 and 116 h post-hCG, and the rate of blastocyst hatching, **(B)** the number of total cells in each blastocyst and **(C)** the number of the cells with fragmented nuclei were analysed. Each culture group had at least 60 hybrid strain embryos and 50 B6 embryos. Statistically significant effects are shown on the graphs. DENSITY and STRAIN were the dependent variables, and INT (abbreviation of interaction) was the interaction effect between DENSITY and STRAIN.

To assess the possibility that severe tropic factor deprivation accounted for the poor development of B6 zygotes we tested the effect of supplementing KSOM plus amino acids media with the known autocrine embryotropin, Paf (37.2 nM). Paf is one of the best characterised autocrine embryotropins and is reported to be limiting during zygote culture [14]. Paf enhanced the rate of blastocyst formation in both strains at 96 h post-hCG (Figure 7A). There was no significant interaction effect between strain and Paf, indicating that the beneficial response to Paf was similar for both strains. At 116 h post-hCG there was no effect of Paf or strain on either the proportion forming blastocysts or those for which hatching had commenced (Figure 7A). The blastocysts formed, however, possessed significantly more cells after Paf treatment (p < 0.001) (Figure 7B) and there was a significant interaction effect between strain and Paf treatments for cell number (p < 0.05), showing that the response to Paf by B6 embryos was greater than for B6CBF1 (Figure 7B). There was no effect of either treatment on the number of nuclei showing degenerate morphology (Figure 7C). This beneficial effect of communal culture or Paf supplementation indicates that under conditions of low oxygen and optimised media design, some deprivation of tropic support for embryos still existed. Yet the extent of this deprivation differed between strains.

**Figure 8:**
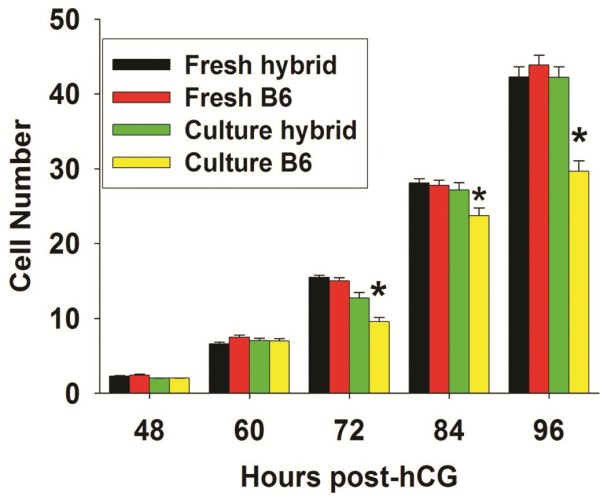
**Testing the effects of optimised culture conditions on embryo growth rates of hybrid strain and inbred strain embryos.** Embryos were collected directly from the reproductive tract during development or zygotes collected and cultured as groups of 10 in 10 μL KSOM media supplemented with amino acids and 37 nM Paf in 5% O_2_ tension. The growth rate of each treatment was assessed by recording the number of cells in each embryo at 12 h interval up to 96 h post-hCG, compared to embryos collected directly from reproductive tract at the same times, in which method was designed as Figure 1. At least 60 embryos for each developmental time point were assessed. There was no differences between in vitro and in vivo hybrid strain embryos at any time point, *p < 0.001 shows the differences in growth rate of B6 embryos in vitro compared to those collected from the reproductive tract.

Next we assessed whether combining all of these variables provided additive growth benefits equivalent to development in the reproductive tract. This experiment was designed as in Figure 1 for both B6 and B6CBF1 using improved conditions (communal culture in KSOM media supplemented with BSA, amino acids and Paf in 5% O_2_). This showed that for both strains the optimised conditions resulted in marked improvements in the rate of accumulation of cells within embryos (Figure 8). The growth rate of hybrid strain embryos was not different to the embryos within the reproductive tract. Yet, despite the marked improvement in the growth of B6 embryos, they were still significantly retarded compared to development in the reproductive tract (P < 0.001). This difference was clearly evident from 72 h post-hCG.

**Figure 9:**
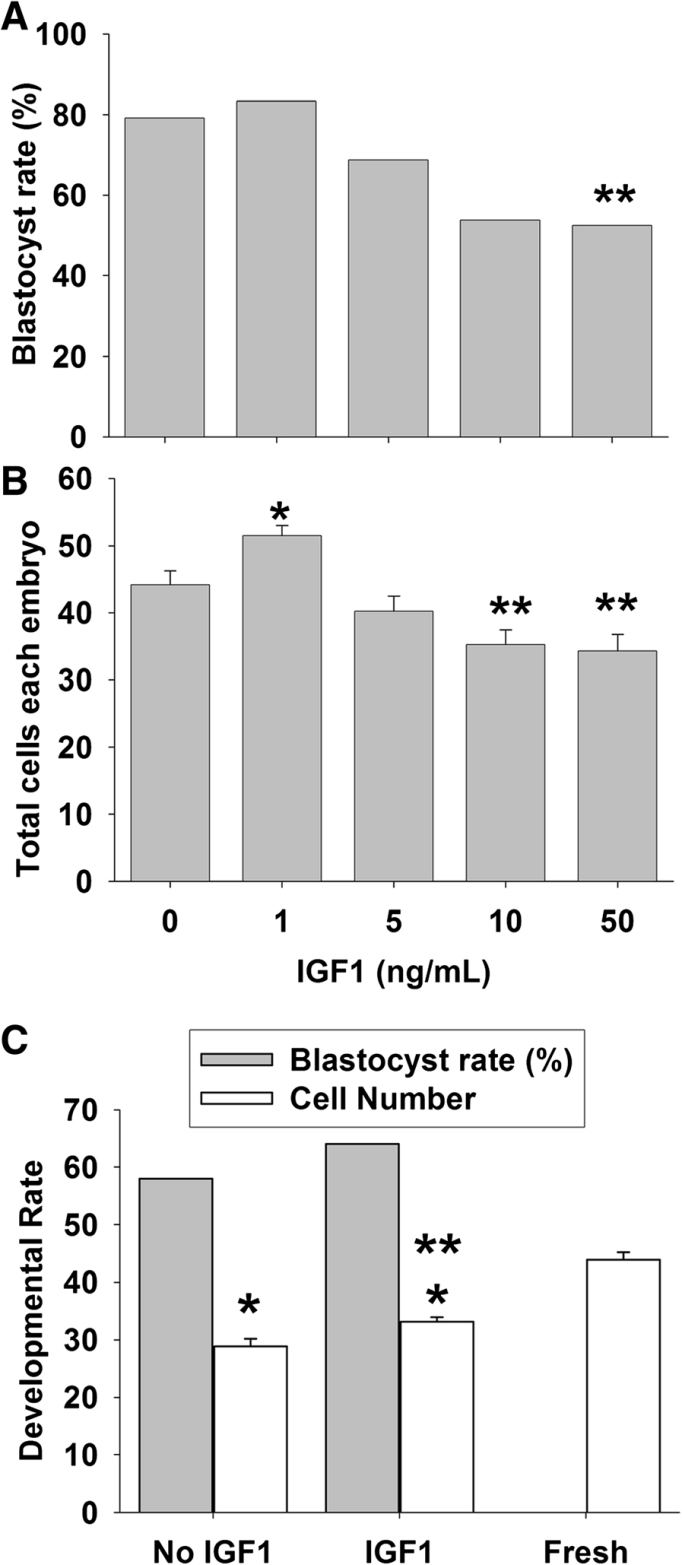
**Effects of IGF1 on B6 embryo development in 5% O**_**2**_**.** B6 zygotes were cultured in groups of 10 μL KSOM media plus amino acid and Paf and supplemented with a range of doses of mouse recombinant IGF1. **(A)** The proportion of zygotes forming blastocysts were assessed 108 h post-hCG. There was an overall adverse dose-dependent effect IGF1 dose on the rate of blastocysts formation P < 0.001, and ** higher dose significant fewer cells (p < 0.01) compared to controls **(B)** total number of cells per blastocyst formed. There was an overall adverse dose-dependent effect of IGF1 (p < 0.001) and multiple comparison of means showed * 1 ng dose to have caused significantly more (p < 0.01) and ** higher dose significant fewer cells (p < 0.01) compared to controls. The results were representative of the three independent replicates with at least 80 embryos each dose. **(C)** The blastocyst rate of B6 zygotes cultured communally in KSOM media plus amino acid and Paf at 5% O_2_ with or without 1 ng/mL IGF1 was shown, and the cell number was compared with blastocysts collected directly from the uterus 96 h post-hCG. *p < 0.001, compared to the fresh embryos. **p < 0.01, compared to the culture conditions without IGF1. The results were representative of three independent replicates and each treatment had at least 90 embryos.

To test if further trophic support could rescue the growth, B6 embryos were cultured in media supplemented with another embryotropin, IGF1. B6 zygotes were cultured in groups in KSOM + amino acids media supplemented with Paf under 5% O_2_ with a dose range of IGF1. There was no effect of low concentrations of IGF1 on the proportion of zygotes forming blastocysts after 108 h, but there was a dose-dependent adverse effect at higher concentrations (10-50 ng/mL) (Figure 9A). A low concentration of IGF1 (1 ng/mL) caused a significant increase in the number of cells accumulated in blastocysts, but this was reversed at higher concentrations (10–50 ng/mL) (Figure 9B). We next compared the growth rates of embryos in media with 1 ng IGF1/mL with development in vivo at 96 h post hCG. This showed that the addition of IGF1 did not affect the blastocyst formation rate, but caused a significant improvement in the number of cells compared to communally cultured zygotes in KSOM + amino acids with Paf under 5% O_2_ (Figure 9C). However, these embryos still had significantly fewer cells (~23%) than those developing in the reproductive tract (Figure 9C).

## Discussion

This study confirms the well established observation that defined media causes a marked retardation of the accumulation of cells in the preimplantation embryo during culture in vitro. This retardation was greatest when culture commenced at the early zygote stage. As expected, culture at an oxygen tension of 5% resulted in less retardation than 20%. Culture in a sequential media design (Sydney IVF media) supported faster development than static media designs (GE-HTF or KSOM), but the addition of mixed amino acids to the static media overcame this difference. In the presence of amino acids no advantage of sequential media design was detected. The benefit of group culture (in hybrid strains) indicated a requirement for autocrine trophic support, and the addition of exogenous trophic ligands provided further additive benefits to growth. The uniformly poor development of B6 embryos (whether cultured individually or communally) and the benefits of exogenous tropic ligands, indicate that embryos of this strain may have a profound deficiency of production of autocrine trophic ligands in vitro. It has been shown that the release of the autocrine embryotrophic ligand (Paf) is under genetic control [22] and this might also apply to other autocrine trophic ligands. The well characterised high level of sensitivity of B6 strain embryos to culture in vitro [19] has been shown to be associated with the loss of P53 regulation by trophic ligands in vitro [25]. This study does not define whether the retarded rates of accumulation of cells within embryos was a result of a reduced rate of cell-cycle progression or an increased rate of cell death (by apoptosis or necrosis) or both. These are important mechanistic questions for future analysis.

A key finding of the study was that while all the variables tested could in isolation provide a growth benefit, none were by themselves able to completely compensate for the retarded cell accumulation rate occurring in vitro. By contrast, the combination of all these beneficial treatments had additive effects so that under the best conditions tested the rate of cell accumulation in hybrid strain preimplantation embryos was not different from the rate observed in the reproductive tract. This suggests that the ionic and nutritional composition of media such as KSOM supplemented with amino acids meet the minimum requires of embryos for optimal cell-cycle progression, provided that they are in a relatively hypoxic environment and received appropriate levels of tropic stimulation, achieved by a combination of communal culture in small media volumes and the addition of exogenous ligands, including Paf and IGF1. It is noteworthy that IGF1 caused a quadratic dose–response with a beneficial effect on cell accumulation at the lowest concentration but this was reversed at the highest concentrations. This is consistent with our earlier observation [14] of the effect of IGF1 on the development of outbred strain embryos, but in that case the dose response was shifted to the right with optimal development occurring at concentrations up to 50 ng/mL and its reversal at 3 μg/mL. A similar quadratic dose-dependent effect has been observed for the effects of Paf on embryos in vitro [26]. It is also important to consider in future if the shape of this dose–response curve is influenced by the co-treatment of embryos with Paf. An important point to be considered is that these potent biological mediators were provided by weight rather than known biological potency. It is probable that such commercial preparations show considerable variability in biological activity between suppliers and batches. An important requirement prior to use of biologicals as part of routine media formulation is to define appropriate standard measures of biological potency and to define the conditions required for stable storage and use of such labile reagents. Failure to do so will result in great variability in outcomes.

While these conditions completely rescued the cell-cycle rate in hybrid strain embryos, it is important to recognise that embryos of this strain are known to be relatively robust in culture and to show a smaller loss of viability than some other strains. So this rescue may not be sufficient to meet the requirements of all embryos in a genetically diverse population that can be expected in a clinical environment. Inbred strains, such as B6, by contrast are recognised for their greater sensitivity to culture and a corresponding loss of viability and developmental potential after culture. This study shows that the culture conditions that were capable of complete rescue of growth parameters of hybrid strain embryos, while providing a large improvement in the growth of B6 embryos, were not sufficient to completely rescue these embryos. The addition of an extra trophic ligand (IGF1) caused further improvement and this may indicate that further screening of a range of target ligands may allow discovery a set of conditions capable of completely rescuing cell accumulation rates across a range of genetic backgrounds.

## Conclusions

The tropic ligands have important roles as survival signals, stimulants of cell-cycle progression [27,28] and may be essential for activation for correct patterns of transcription from the early embryo at the time of embryonic genome activation [29]. The narrow effective dose range of trophic ligands (and their adverse effects at supraphysiological levels), however, indicates that caution is required in the clinical use of such agents [30]. There should be no rush to the clinic with their use without appropriate thorough investigation of their effectiveness and safety. This study shows that the use of culture-sensitive mouse strains, such as B6, may be valuable tools for further identification of the conditions and treatments required for the ongoing optimisation of embryo culture techniques.

## Competing interests

The authors declare that they have no competing interests.

## Authors’ contributions

CO conceived and designed the study. XLJ performed the experiments and data analysis. CO and XLJ prepared the manuscript. Both authors read and approved the final manuscript.
